# Long-Term Retrospective Analysis of Re-do Microvascular Decompression in Patients With Hemifacial Spasm

**DOI:** 10.3389/fneur.2021.687945

**Published:** 2021-09-01

**Authors:** Jiayu Liu, Fang Li, Guangyong Wu, Bo Liu, Jingru Zhou, Cungang Fan, Feng Jiao, Dongliang Wang, Gang Wu, Haidong Song, Ruen Liu

**Affiliations:** ^1^Department of Neurosurgery, Peking University People's Hospital, Beijing, China; ^2^Department of Neurosurgery, The Hospital of Shunyi District, Beijing, China

**Keywords:** hemifacial spasm, persistence, recurrence, microvascular decompression, conflict zone

## Abstract

**Objective:** To explore the clinical characteristics of patients with persistent or recurrent hemifacial spasm (HFS) and the experience of microvascular decompression (MVD) in the treatment of such patients to accumulate additional clinical evidence for optimal treatment protocols.

**Methods:** We retrospectively analyzed the clinical data, surgical methods and treatment efficacies of 176 patients with persistent or recurrent HFS from January 2009 to January 2018.

**Results:** Missing compression zones was the main reason for symptom persistence (87.50%) or recurrence (71.50%) after MVD treatment of HFS. We divided the surgical area into three zones. Most persistent or recurrent cases had decompression only in the root exit zone (REZ) (Zone 1) but missed the ventrolateral pons-involved area (Zone 2) or the bulbopontine sulcus-involved area (Zone 3) in the first MVD. Too much use of Teflon (12.50%), arachnoid adhesions (5.60%) and Teflon granulomas (10.40%) can also cause a recurrence. The difference between preoperative and postoperative Cohen scores was statistically significant in persistent or recurrent HFS patients (p<0.05). The postoperative follow-up time ranged from 36 to 108 months (71.75 ± 22.77).

**Conclusions:** MVD should be performed in the compression site, which is mostly located at the brainstem/facial REZ. Intraoperative exploration should be conducted in accordance with the abovementioned zones to effectively avoid missing offending vessels. Re-do MVD is effective in patients with persistent or recurrent HFS.

## Introduction

Primary hemifacial spasm (HFS) mainly manifests as parabolic involuntary twitching of one side of the facial muscles, which can lead to small eyelid fission and crooked mouth corners in some patients, thereby seriously affecting their quality of life (1). Neurovascular compression (NVC), proposed by Jannetta, which causes short circuits of nerve conduction due to microvascular compression, is currently recognized by most scholars as the pathogenesis of primary HFS (2). Microvascular decompression (MVD), based on Jannetta's peripheral nerve theory, has been proven to be effective (3). Although the cure rate of MVD for HFS is 70 to 98% (4), there is a 3.3 to 20% recurrence rate within five years after the operation (5). The purpose of this study was to explore the clinical characteristics of patients with persistent or recurrent HFS and the use of MVD in the treatment of such patients to accumulate additional clinical evidence for optimal treatment protocols.

## Materials and Methods

### Patients

Clinical data from 176 patients with persistent or recurrent HFS after prior failure of MVD were collected between January 2009 and January 2018 at the Department of Neurosurgery, Peking University People's Hospital, the Seventh Medical Center of PLA General Hospital and Characteristic Medical Center of Strategic Support Force. Recurrent HFS was defined as the resurgence of facial spasms on the same side after a previous successful MVD with complete symptom relief without any medication. Re-do MVD was carried out by the corresponding author, Ruen Liu. Written informed consent was obtained from each participant, and the study was approved by the institutional review board of the hospitals.

### Preoperative Management

All patients underwent preoperative MRI, including 3D T1- and T2-weighted high-resolution sequences, to clearly display the trigeminal nerve and all vascular structures. Using three-dimensional time-of-flight magnetic resonance angiography (MRA) allows for the display of high-flow blood vessels, mainly arteries.

### Operative Technique

**Figure 1:**
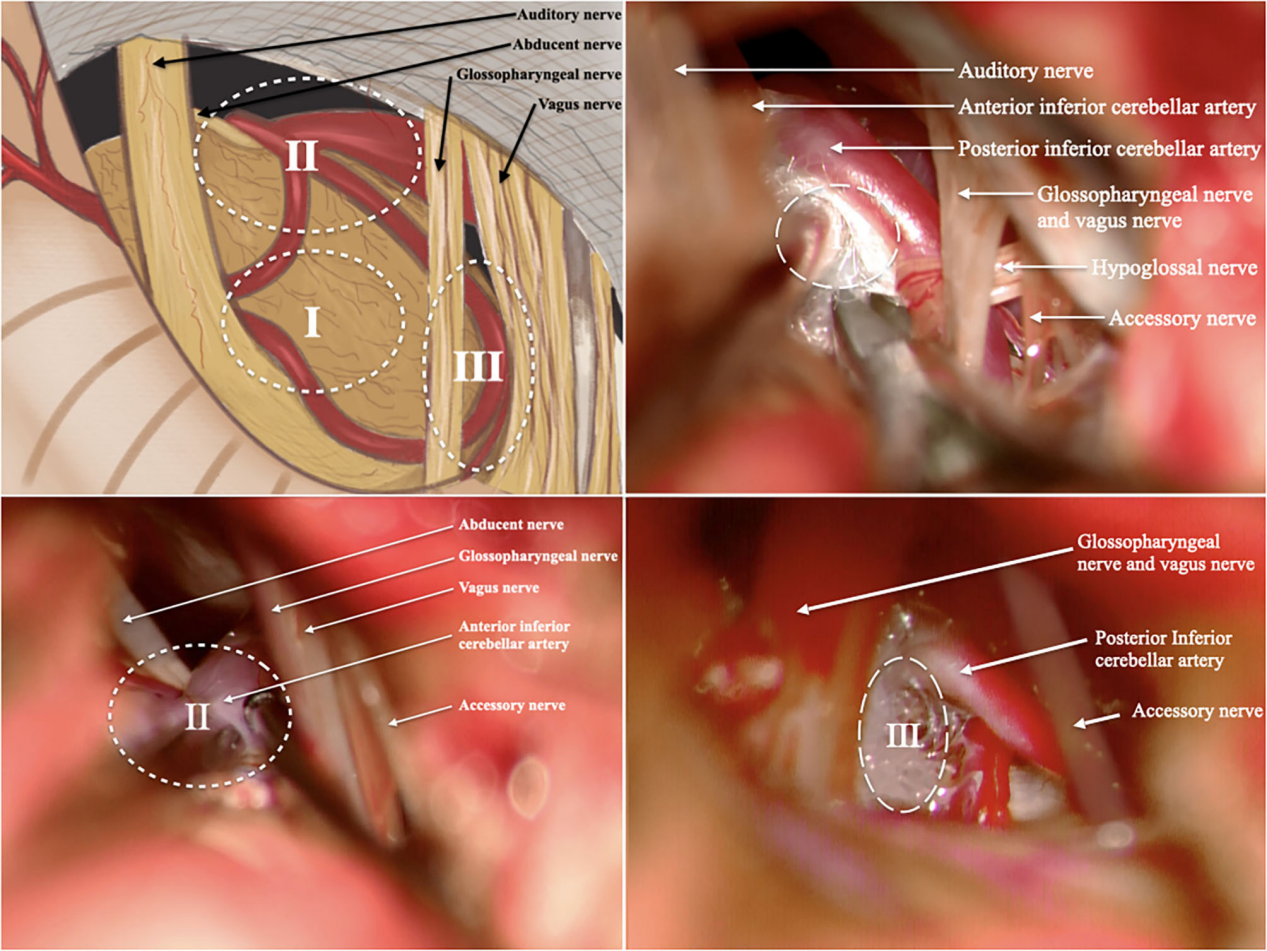
We divided the surgical area into 3 zones. **(A)** Illustration of the surgical area; **(B)** illustration of Zone 1; **(C)** illustration of Zone 2; **(D)** illustration of Zone 3; (I, Zone 1 is involved in the facial nerve and root exit zone (REZ); II, Zone 2 is involved in the ventrolateral pons; III, Zone 3 is involved in the bulbopontine sulcus). All patients in these pictures have right side HFS. The rostral is on the left and the caudal is on the right.

The preoperative management and operative technique were consistent with our previous studies (1, 6). After the induction of general anesthesia, the patient was placed in the lateral park bench position with three-point fixation, and retrosigmoid craniotomy was performed. We divided the surgical area into three zones: Zone 1 was involved in the facial nerve and root exit zone (REZ), Zone 2 was involved in the ventrolateral pons (the area where the abducens nerve intersects with the anterior inferior cerebellar artery), and Zone 3 was involved in the bulbopontine sulcus (Figure 1). First, the previous NVC area was checked again to confirm that satisfactory decompression was achieved. Tracing the offending vessel began from zone 1 to zone 3. For patients whose decompression zone was missed in the first MVD, we inserted a prosthesis between the offending vessels and the affected nerve to separate the nerve-vessel conflict. Teflon material is felt substance, and its shape is balls. We interposed the Teflon between the facial nerve and vascular. The Teflon is touching the nerve. For patients with too much use of Teflon in the first MVD, the material was dissected, and part of it was removed to a proper thickness. If the nerve was distorted by the presence of arachnoid adhesions or a Teflon granuloma, the nerve was freed via careful dissection. In addition, monitoring of brainstem auditory evoked responses and motor evoked potentials of the facial nerve was performed during the surgery by an experienced neurophysiological monitoring team. Ephaptic impulse transmission along the facial nerve must be obliterated in order to confirm an adequate decompression. Ephaptic transmission is judged to be eliminated once the “lateral spread” of impulses from one motor branch of the facial nerve to another is gone and cannot be elicited despite intraoperative facial nerve stimulation (7).

### Data Collection

Baseline data and medical history information were obtained from the patients' medical records. The baseline data included age at re-do MVD, sex, history of hypertension, duration of symptoms, interim to re-do MVD, causes of recurrence, and preoperative and postoperative status based on the Cohen evaluation scale (8). The Cohen evaluation scale has a score of 0–4, with 0 = none; 1 = increased blinking caused by external stimuli; 2 = mild, noticeable fluttering, not incapacitating; 3 = moderate, very noticeable spasm, mildly incapacitating; and 4 = severely incapacitating (unable to drive, read, etc.).

### Statistical Analysis

In this study, SPSS statistical software 19.0 (IBM Corp., Armonk, NY, USA) was used for the data analysis. Numerical variables are expressed in terms of the mean ± standard deviation (mean ± SD). Categorical variables are expressed as frequencies (%). We used Student's *t*-test to evaluate the data that followed a normal distribution. *P* < 0.05 was considered a significant difference between groups.

## Results

### Baseline Characteristics

Of the 176 patients, 144 had recurrent disease, and 32 had persistent disease. Only 10 recurrent patients underwent their first MVD at our hospital.

A total of 144 recurrent cases (47 men, 97 women) were included, and the age of the participants ranged from 34 to 85 years (60.44 ± 11.38). All patients had unilateral symptoms, and 51 cases (35.42%) were affected on the left side. The duration of HFS ranged from 2 to 10 years (6.42 ± 2.21). There were 36 patients (25.00%) with a history of hypertension. The interval to repeat MVD ranged from 1 to 6 years (2.85 ± 1.55). The preoperative Cohen scores were 4 for 27 patients (18.80%) and 3 for 117 patients (81.30%) (Table 1).

**Table 1:** Clinical characteristics of all patients with recurrent hemifacial spasm.

**Characteristic**	**Recurrent patients(*n* = 144)**	**Persistent patients (*n* = 32)**
Age -yr	60.44 ± 11.38	60.10 ± 12.40
Female sex – no. (%)	97 (67.40%)	19 (29.38%)
Left side – no. (%)	51 (35.42%)	21.90%
History of Hypertension – no. (%)	36 (25.00%)	10 (31.30%)
duration of symptoms -yr	6.42 ± 2.21	6.00 ± 2.34
Interim to re-do MVD -yr	2.85 ± 1.55	2.69 ± 1.20
**Failure reason of the 1st MVD – no. (%)**		
Missed depression zone	103 (71.50%)	28 (87.50%)
Too much use of Teflon	18 (12.50%)	4 (12.50%)
Arachnoid adhesion	8 (5.60%)	-
Teflon Granuloma	15 (10.40%)	-
**Preoperative Cohen score – no. (%)**		
3	117 (81.30%)	7 (21.90%)
4	27 (18.80%)	25 (78.10%)
**Postoperative Cohen score – no. (%)**		
0	109 (75.69%)	27 (84.40%)
1	20 (13.89%)	3 (9.40%)
2	4 (2.78%)	2 (6.20%)
3	5 (3.47%)	-
4	6 (4.17%)	-

A total of 32 persistent cases (13 men, 19 women) were included, and the age of the participants ranged from 24 to 84 years (60.10 ± 12.40). All patients had unilateral symptoms, and 7 cases (21.90%) were affected on the left side. The duration of HFS ranged from 2 to 10 years (6.00 ± 2.34). There were 10 patients (31.30%) with a history of hypertension. The interval to repeat MVD ranged from 1 to 5 years (2.69 ± 1.20). The preoperative Cohen scores were 4 for 7 patients (21.90%) and 3 for 25 patients (78.10%) (Table 1).

### Surgical Findings in the Recurrent Patients

In 144 recurrent patients, there were 103 patients (71.50%) whose decompression zone was missed in the first MVD. These patients had satisfactory decompression of Zone 1 during the first MVD. However, a conflict site was discovered in the re-do MVD, although it was caused by the same offending artery. Of these, in 85 patients (82.53%), the anterior inferior cerebellar artery (AICA) was identified in Zone 2, which was regarded as the offending vessel in the above conflict site. In 18 patients (17.47%), the offending vessels observed were the AICA, posterior inferior cerebellar artery (PCIA) and AICA combined with PICA in Zone 3.

In 18 patients (12.50%), the cause of recurrence was too much use of Teflon in the first MVD. Of these, 12 patients (66.70%) had compression in Zone 1, and 6 patients (33.30%) had compression in Zone 2. The offending vessels observed were AICA.

Finally, an arachnoid adhesion or a Teflon granuloma in Zone 1 was found in eight patients (5.60%) and 15 patients (10.40%), respectively.

### Surgical Findings in the Persistent Patients

In 32 persistent patients, there were 28 patients (87.50%) whose decompression zone was missed in the first MVD. Of these, in three patients (10.71%), AICA was identified in Zone 1, which was regarded as the offending vessel in the above conflict site. These patients had the Teflon inserted between the AICA and the facial nerve trunk during the first operation rather than the REZ, and we believe this is the reason why their first operation failed. In the other 25 patients (89.29%), the offending vessels observed were PCIA in Zone 3.

In the 4 persistent patients (12.50%), the cause of the persistence was too much use of Teflon in the first MVD in Zone 1.

### Outcomes of Re-do MVD

In recurrent patients, the re-do operation failed in two patients due to a large looped vertebral artery. The postoperative Cohen scores were 0 for 109 patients (75.69%), 1 for 20 patients (13.89%), 2 for 4 patients (2.78%), 3 for 5 patients (3.47%) and 4 for 6 patients (4.17%). The difference between the preoperative and postoperative Cohen scores was statistically significant (*p* < 0.05). Delayed relief was found in eight patients. It took within 2 weeks for them to obtain complete relief. Other patients were cured immediately after the redo MVD.

In persistent patients, the postoperative Cohen scores were 0 for 27 patients (84.40%), 1 for 3 patients (9.40%), and 2 for two patients (6.20%). The difference between the preoperative and postoperative Cohen scores was also statistically significant (*p* < 0.05). Delayed relief was found in two patients. It took 6 days and 13 days for them to obtain complete relief. Other patients were cured immediately after the redo MVD.

Each group had no mortality, no intracranial haematoma, no complete hearing loss, or other serious complications. Two patients (1.14%) had complete facial paralysis, 15 patients (8.52%) had partial hearing loss, 17 patients (9.66%) had transient facial paralysis, 8 patients (4.55%) had scalp tingling, 3 patients (1.70%) had leakage of cerebrospinal fluid, and 3 patients (1.70%) had transient vertigo. Except for complete facial paralysis, all complications were cured by symptomatic and supportive treatment.

The postoperative follow-up time ranged from 36 to 108 months (71.75 ± 22.77). In the follow-up period, five patients with a Teflon granuloma found in the re-do MVD group had recurrent symptoms and did not undergo MVD again.

## Discussion

Hemifacial spasm (HFS) is a cranial nerve disease characterized by recurrent, involuntary and painless twitching of the muscles innervated by one side of the facial nerve (1). They are most common in women around the age of 50 and are mainly unilateral, which is consistent with the results of this study. Since Jannetta first systematically elaborated the theory of MVD in 1975, MVD has gradually been accepted by most neurosurgeons and it has become the main treatment for HFS (3). Although the cure rate of MVD for HFS is 70 to 98% (4), there is a 3.3 to 20% recurrence rate within 5 years after surgery (5). Recurrence after MVD treatment of HFS is defined as the recurrence of symptoms after a period of complete recovery, which may be lighter, more severe, or the same as before (16). Due to the demyelination of the facial nerve site, which is compressed by the responsible blood vessels, and the excitability of the facial nerve nucleus, it takes some time to recover after surgery (17). Kondo et al. (18) defined recurrence of symptoms within 1 year as uncured and recurrence after 1 year as a recurrence. The suitable time for recurrent HFS patients after their first MVD to receive MVD again is under debate (10). However, most studies suggest that the treatment of recurrent HFS should be more than 1 year after the previous MVD (19, 20). In this study, we performed reoperations on patients whose interval from their prior MVD was >1 year (21). Patients with prior MVD elsewhere did not have adequate information about their prior MVD. However, combined with the clinical data and follow-up results of this group of patients, we believe that the main reasons for symptom persistence or recurrence after MVD treatment of HFS are as follows: depression zone missed, too much use of Teflon, arachnoid adhesion and Teflon granuloma.

In our study, missed depression zones were the main cause of HFS persistence or recurrence. Misjudgement or omission of responsible vessels is the main reason for this (9–15) (Table 2). For this reason, we believe that familiarity with the microanatomy of the cerebellopontine angle region, correct identification of responsible vessels and effective decompression of nerve roots are important guarantees to improve the surgical effect and reduce the risk of recurrence. Good surgical habits are the most important factors influencing the surgical outcome (22).

**Table 2:** The data from literature relating the causes of failures/recurrences.

**References (N)**	**anatomical causes of failures/recurrences**	**Percentage**
Engh et al. (9) (36)	Aborted MVD	19.4% (7/36)
	Missed compression	58.3% (21/36)
	Too much use of Teflon	8.3% (3/36)
	Arachnoid adhesion	5.6% (2/36)
	Unknown	8.3% (3/36)
Bigder et al. (10) (12)	Missed compression	91.7%(11/12)
	aneurysm arising	8.3%(1/12)
Jiang et al. (11) (26)	Missed compression	57.7% (15/26)
	Too less use of Teflon	38.5% (10/26)
	Too much use of Teflon	3.8% (1/26)
Lee et al. (12) (21)	Missed compression	66.7% (14/21)
	Too less use of Teflon	33.3% (7/21)
Xu et al. (13) (78)	Missed compression	71.8% (56/78)
	Too less use of Teflon	20.5% (16/78)
	Too much use of Teflon	10.3% (8/78)
	Arachnoid adhesion	9.0% (7/78)
Ravina et al. (14) (8)	Missed compression	62.5% (5/8)
	Teflon granuloma/ adhesion	25.0% (2/8)
	Too less use of Teflon	12.5% (1/8)
Park et al. (15) (23)	Missed compression	52.2% (12/23)
	Too less use of Teflon	34.8% (8/23)
	Teflon adhesion	13.0% (3/23)

We divided the surgical area into three zones: Zone 1 was involved in the facial nerve and root exit zone (REZ), Zone 2 was involved in the ventrolateral pons (the area where the abducens nerve intersects with the anterior inferior cerebellar artery), and Zone 3 was involved in the bulbopontine sulcus. In our study, most depression zone missed cases had decompression in Zone 1 (REZ area) but missed Zone 2 or Zone 3 in the first MVD. The offending vessels usually form a loop through the facial nerve REZ and cause compression. When there are multiple vessels in the REZ, the offending vessels are usually located in the deep side of the vascular plexus. In addition, decompression is often difficult when the responsible vessels are thick or tortuous, there are multiple short perforating arteries, or the perforating arteries are located only in the facial and acoustic nerve roots. Therefore, in the process of MVD, it is not enough to perform responsible vascular exploration and decompression only in the REZ region (Zone 1). In addition to the REZ region, neurosurgeons should also pay attention to the other two regions in MVD. Intraoperative exploration should be conducted in accordance with the abovementioned zones to effectively avoid missing offending vessels. Furthermore, MVD should be performed in the compression site, which is mostly located at the brainstem/facial REZ (23). In this study, we found that three persistent patients had Teflon inserted between the AICA and the facial nerve trunk during the first operation rather than the REZ, and we believe this is the reason why their first operation failed.

Improper use of Teflon is another cause of HFS persistence or recurrence. In MVD, we believe that Teflon can play three roles: (1) isolate responsible blood vessels from the brainstem/facial REZ, (2) insulate abnormal nerve conduction caused by compression of responsible blood vessels, and (3) absorb the kinetic energy generated by the pulse of responsible blood vessels. Excessive decompression is due to the fear of insufficient transportation of the responsible vessels, and too much Teflon is placed between the facial nerve root and the responsible vessels. Subsequently, a local inflammatory reaction occurs, and the hyperplasia and wrapping of the arachnoid makes the Teflon cotton directly compress the facial nerve root, leading to recurrence.

In addition, insufficient decompression can also cause HFS recurrence. It was speculated that the soft Teflon cotton was inserted during the operation, which displaced the responsible vessels, and the decompression was sufficient. However, under the continuous pulsation of the blood vessels, the soft Teflon cotton gradually flattened and became thin, and the responsible vessels moved back, causing compression again. Therefore, the Teflon should be inserted appropriately between the brain stem and the responsible blood vessels to avoid the possible formation of new compression or even compression of the brain stem, resulting in complications; alternatively, if too little Teflon is inserted, the pulsating shock of the responsible vessels can still be transmitted to the facial nerve REZ. We believe that separating the NVC is the most suitable thickness for Teflon, and we should insert the Teflon instead of pushing it in to avoid moving back the responsible vessels that were pushed away and causing the recurrence. For patients with too much use of Teflon in the first MVD, the material was dissected, and part of it was removed to a proper thickness. Except in cases using too much Teflon or a Teflon granuloma, it is not necessary to remove the Teflon completely because the Teflon pads attach to some small blood vessels and nerve fibers during the separation process, which can easily cause tension and injury.

In this study, arachnoid adhesion was also found to be one of the causes of recurrence. The arachnoid abnormality in the outer layer of the CPA restricted the trigeminal nerve root by arachnoid bonding. Although vascular compression is relieved in some MVD surgeries, such arachnoid bonds still exist. Ishikawa et al. (24) reported that in 53 cases of MVD, there were nine cases without responsible blood vessels, and the effect of periarachnoid separation during the operation was positive. Thickened arachnoid bonds restrict the movement of nerve roots. Symptoms are caused by an increased pulsating force and peripheral compression due to the fixation of the nerve roots during pulsation of the cerebrospinal fluid and surrounding blood vessels. Therefore, adequate intraoperative arachnoid separation is an important measure to improve the efficacy and prevent postoperative recurrence. Surgical injury caused by subarachnoid hemorrhage and arachnoid edge adhesion causes local cerebrospinal fluid circulation disorders and even the formation of arachnoid cysts (25). Recurrence occurs when a pair of arachnoid cysts containing large amounts of cerebrospinal fluid form, causing direct compression of nerves, or arterial pulsation through the arachnoid cyst causes compression of the nerve. In addition, re-do MVD will be more difficult because of local arachnoid adhesion and unclear anatomical relationships and must be carefully performed during the operation to avoid complications such as hearing loss and facial paralysis.

Finally, Teflon granuloma is also a cause of recurrent HFS. In our previous study of 127 cases of recurrent trigeminal neuralgia, 23 cases were found to be caused by a granuloma (6). In this study, we also found the same situation in HFS patients. Teflon granulomas can attach to blood vessels, nerves, or the brain stem, causing nerve distortion or even compression and eventually causing a recurrence of symptoms (26). Neuroimaging studies are helpful in the diagnosis of granulomas, which often appear on T2-weighted images as low-intensity contrast accumulation areas with mass effects (27). The mechanisms of their formation are not clear and may be related to local chronic inflammation around the Teflon. The presence of haemosiderin-containing macrophages in the histological study of granulomas suggests that small intraoperative bleeding into the Teflon may contribute to granuloma formation (28). The immune cells then aggregate in the Teflon, forming chronic granulomatous inflammation. Therefore, avoiding blood flow into the Teflon during surgery is an effective and important measure to prevent granuloma formation. On the other hand, whether the use of some material for fixation of the Teflon is a risk factor for granuloma development remains unclear (28). However, we believe that avoiding unnecessary substances, such as bone wax, falling into the Teflon can also reduce the incidence of postoperative granulomas. If NVC is found in a re-do MVD, it is safe to place new Teflon because the removal of granulomatous masses and adhesions prevents inflammation (28).

Our study demonstrated that re-do MVD is effective in patients with persistent or recurrent HFS. However, we also found the occurrence of partial hearing loss, transient facial paralysis and other complications. Payner et al. (29) reported that the occurrence of hearing impairment after secondary MVD in HFS was related to intraoperative injury of the auditory nerve and/or its nourishing vessels. The second operation will be more difficult because of local arachnoid adhesions and unclear anatomical relationships. The surgical time required to explore the facial nerve is relatively long, and the cerebellum and facial nerve receive more mechanical stimulation. When the cotton pads are separated from the nerve or the blood vessels are attached to the nerve, the probability of nerve injury is increased. Great care should be taken during the operation to avoid complications such as hearing loss and facial paralysis.

## Conclusion

In summary, this study explored the clinical characteristics of patients with persistent or recurrent HFS and the experience of MVD in the treatment of such patients to accumulate additional clinical evidence for optimal treatment protocols. We divided the surgical area into three zones. Most persistent or recurrent cases had decompression in the REZ (Zone 1) but missed Zone 2 or Zone 3 in the first MVD. MVD should be performed in the compression site, which is mostly located at the brainstem/facial REZ. Intraoperative exploration should be conducted in accordance with the abovementioned zones to effectively avoid missing offending vessels. Too much Teflon, arachnoid adhesions and Teflon granulomas were also reasons for symptom recurrence after MVD treatment of HFS. Teflon should be inserted appropriately between the brain stem and responsible blood vessels. Avoiding blood flow into the Teflon during surgery is an effective and important measure to prevent granuloma formation.

## Data Availability Statement

The raw data supporting the conclusions of this article will be made available by the authors, without undue reservation.

## Ethics Statement

The studies involving human participants were reviewed and approved by the institutional review board of Peking University People's Hospital. The patients/participants provided their written informed consent to participate in this study.

## Author Contributions

JL: literature search, study design, data collection, data interpretation, and writing. RL and FL: study design and provided feedback on all manuscript texts. GW, BL, JZ, CF, FJ, DW, GW, and HS: data collection and provided feedback on all manuscript texts. All authors contributed to the article and approved the submitted version.

## Conflict of Interest

The authors declare that the research was conducted in the absence of any commercial or financial relationships that could be construed as a potential conflict of interest.

## Publisher's Note

All claims expressed in this article are solely those of the authors and do not necessarily represent those of their affiliated organizations, or those of the publisher, the editors and the reviewers. Any product that may be evaluated in this article, or claim that may be made by its manufacturer, is not guaranteed or endorsed by the publisher.
